# Pleistocene Climate, Phylogeny, and Climate Envelope Models: An Integrative Approach to Better Understand Species' Response to Climate Change

**DOI:** 10.1371/journal.pone.0028554

**Published:** 2011-12-02

**Authors:** A. Michelle Lawing, P. David Polly

**Affiliations:** 1 Department of Geological Sciences, Indiana University, Bloomington, Indiana, United States of America; 2 Department of Biology, Indiana University, Bloomington, Indiana, United States of America; University of Copenhagen, Denmark

## Abstract

Mean annual temperature reported by the Intergovernmental Panel on Climate Change increases at least 1.1°C to 6.4°C over the next 90 years. In context, a change in climate of 6°C is approximately the difference between the mean annual temperature of the Last Glacial Maximum (LGM) and our current warm interglacial. Species have been responding to changing climate throughout Earth's history and their previous biological responses can inform our expectations for future climate change. Here we synthesize geological evidence in the form of stable oxygen isotopes, general circulation paleoclimate models, species' evolutionary relatedness, and species' geographic distributions. We use the stable oxygen isotope record to develop a series of temporally high-resolution paleoclimate reconstructions spanning the Middle Pleistocene to Recent, which we use to map ancestral climatic envelope reconstructions for North American rattlesnakes. A simple linear interpolation between current climate and a general circulation paleoclimate model of the LGM using stable oxygen isotope ratios provides good estimates of paleoclimate at other time periods. We use geologically informed rates of change derived from these reconstructions to predict magnitudes and rates of change in species' suitable habitat over the next century. Our approach to modeling the past suitable habitat of species is general and can be adopted by others. We use multiple lines of evidence of past climate (isotopes and climate models), phylogenetic topology (to correct the models for long-term changes in the suitable habitat of a species), and the fossil record, however sparse, to cross check the models. Our models indicate the annual rate of displacement in a clade of rattlesnakes over the next century will be 2 to 3 orders of magnitude greater (430-2,420 m/yr) than it has been on average for the past 320 ky (2.3 m/yr).

## Introduction

The Intergovernmental Panel on Climate Change reported that mean annual temperature will increase anywhere from 1.1 to 6.4°C by the end of the 21^st^ century [1]. A shift in temperature as large as 6°C is comparable to the mean annual temperature difference between a glacial and an interglacial climate [2]. Many species, especially in temperate regions, shift their geographic distributions dramatically between glacial and interglacial cycles. These orbitally-forced geographic distribution dynamics influence speciation, range sizes, and latitudinal patterns [3]. Further, many species have significantly shifted their geographic distributions in the past few decades [4], [5]. If a species is not able to track its suitable habitat or adapt to these changing climatic conditions, it will become extinct [6].

The response of species to climate change has often been studied in field settings by tracking a species' geographic movement through years or decades. In a meta-analysis of 1,700 species compiled from published field studies, habitat tracking poleward is on average 0.61 km yr^−1^ [7]. This approach provides a good estimate of the current rate of geographic displacement in response to climate change; however, it does not provide a framework for comparison with a background rate of change (an expected rate). An expectation can be established from estimating past rates of change and is an important indicator as to whether the current response is within the range of normality and if it is sustainable. Here we extend existing methods so that we can model suitable habitats for species in the geological past through a series of glacial-interglacial cycles. Our aim is to make use of the rich, continuous record of changes in global climate through the Quaternary that has emerged from stable isotope data and paleoclimate modeling. By using stable oxygen isotope data, we have derived a geographically explicit climate data set for the entire late Quaternary of North America by scaling between two general circulation climate models (GCMs) for glacial and interglacial periods. These data provide us with a set of North American paleoclimate maps for the last 300,000 years spaced ∼4,000 years apart onto which we have projected phylogenetically scaled climate envelopes to estimate the geographic distributions of a clade of eleven rattlesnakes. The rates of historical waxing and waning of habits through several glacial-interglacial cycles provides a comprehensive non-anthropogenic context for evaluating current rates of geographic displacement due to anthropogenic climate change.

A climate envelope model generally characterizes a set of suitable habitats for a species derived from their present geographic location. The climate envelope models are constructed from the associations between the geographic position of a species' occurrence and its climate. Climate envelope models can be selected, trained, and tested under current climate conditions [8], but there is difficulty in testing these models under different climates. Insight can be gained from comparing projections of climate envelope models on paleoclimate maps with fossil occurrences [9], from projecting climate envelope models of invasive species onto continents that are being invaded [10], [11], or from comparing projections of climate envelope models on paleoclimate maps with traditional molecular genetic phylogeographic predictions of potential distributional areas [12].

The climate envelope model has been critiqued for disregarding biotic interactions, dispersal, and evolutionary change [13], [14], [15]. In addition, climate envelope modeling takes into account species' occurrences, which only represent their realized niche. The realized niche is the current space occupied by a species resulting from biotic and abiotic interactions, where the fundamental niche is all possible space that supports viable populations within a species, but in which the species does not necessarily currently occupy [16]. Review of these criticisms suggests climate envelope modeling does provide a useful first approximation to study the dynamics of species' response to climate change when considered at the appropriate macro scale [17]. In addition, a study comparing climate envelope models and mechanistic models in 100 plant species suggest some climate envelope models can be used to predict changes in a species' geographic distribution under alternative climate scenarios [18].

An alternative approach to modeling suitable habitat for a species is mechanistic modeling [19], [20], [21]. A mechanistic model is based on the functional morphology, behavior and physiological requirements of a species and is independent of the species' geographic distribution and the climate in which the species lives [22]. These functional traits are then linked to climate and environmental variables, which can be mapped in geographic space. Functional trait data are required as input parameters in mechanistic models and specifically physiological data, such as temperature tolerances and energy, water, and mass balance, are not always readily available for species being studied. Identifying species' physiological requirements involves experimentation that is costly, time consuming, and impractical when dealing with many species.

Another aspect of climate envelope modeling concerns how to take phylogenetic effects into account. Phylogenetic comparative methods (PCM) have been used to account for phylogenetic relatedness and to elucidate past rates of change of climate tolerances by reconstructing ancestral climate envelopes [23], [24], [25]. The PCM approach models changes in the climate envelope of a species, without necessarily projecting those changes into geographic space by use of paleoclimate maps. By projecting the reconstructed envelope onto geographic space, the past location and size of the ancestor's suitable habitat can be estimated, but a contemporaneous paleoclimate map is needed, something that has heretofore been unavailable to PCM-based studies. We extend the PCM approach by modeling evolving envelopes along the branches of the phylogenetic tree, not just the ancestral nodes, and we project the evolving envelopes onto the series of paleoclimatic maps that we generate for every temporal step along the branch using the oxygen isotope data and GCM models.

Previous studies have estimated past geographic distributions at 6, 21, and 126 kya by projecting the climate envelopes of extant species' models onto GCMs for those times (reviewed in [26]). These snapshots show how suitable habitats of species are likely to have shifted geographically with climate change, assuming no evolution. However, several studies indicate that climatic niches of species can evolve over short evolutionary time scales [27], [28], [29], which suggests past climate envelope modeling that takes into account phylogeny might be more informative than those that do not. The further back in time a climate envelope model is extrapolated, the more critical it is to account for evolutionary adaptation in its construction [23], [30].

GCMs are complex integrations of mathematical functions that describe atmospheric and oceanic circulation, sea ice, land surface properties, and atmospheric properties. GCMs use computationally intensive numerical models to simulate past, present, and future climates, including atmospheric and oceanic temperature and precipitation. Because of the computationally intensive nature of GCMs, only a limited set of past global climates have been modeled. Examples of such paleoclimate models for the Quaternary are for 0, 6, and 21 kya, produced by the Paleomodelling Intercomparison Project II [31], and for the last interglacial ∼120–140 kya by Otto-Bliesner et al. [32].

In contrast, nearly continuous high-resolution estimates of global and local mean annual paleotemperatures are available for many parts of the world in the form of oxygen isotope values from sediment and ice cores for the entire Quaternary, and even the entire Cenozoic [33], [34]. In order to estimate temperature and precipitation for past climates that may not have been modeled with GCMs, we use stable oxygen isotope values to interpolate between two time periods when climate is either known (as in the present) or has been modeled. We used this interpolation to construct paleoclimate maps at ∼4 ky time intervals for the last 320 ky.

Here we developed phylogenetically-informed climate envelope models for 11 rattlesnake species (*Crotalus*). We project a phylogenetically reconstructed climate envelope onto a map of paleoclimate at many corresponding time intervals over the last 320 ky to determine the potential rate of geographic change in a species' suitable habitat. We refer to these models as paleophylogeographic models. By synthesizing both the evolutionary history of climatic tolerances and the climatic history in which these species evolved, we have arrived at a more detailed understanding of how they have responded to climate change and we predict how they might respond in the future.

## Materials and Methods

### Study System

Snakes are particularly useful for understanding the effects of climate change on terrestrial vertebrate species because their ectothermic physiology is highly dependent on the ambient temperature. We specifically chose rattlesnakes because the geographic distributions of some species extend north of former glacial margins, assuring that their geographic distributions have, in fact, changed over recent geological history [35]. The genus *Crotalus* originated in North America, most likely in the mid-Miocene, 13–15 mya [36], and its subsequent history on the continent is a complicated combination of dispersal, vicariance, and speciation in the context of changing climate and geography [37], [38], [39], [40].

### Species' Occurrences

Detailed geographic distributions of 11 rattlesnake species were obtained from published range maps [35]. The sampling scheme of Polly [41] was adopted, which is essentially a point based scheme (available at http://mypage.iu.edu/~pdpolly/Data.html). First, points spaced by 50 km were laid across North America and then points that overlap with a species' geographic distribution were selected to represent that species' geographic distribution. We call these range occurrences. We chose to base our models on an evenly spaced set of occurrence points sampled using equidistant 50 km points across each modern species' geographic distribution, instead of point occurrence data derived from museum collection events, such as the Global Biodiversity Information Facility, www.gbif.org, because our 11 species are poorly covered, are commonly misidentified in museum collection records or existing collection records have a strong geographic bias, especially in Mexico. Geographic distribution maps of these species [35], primarily stemming from sightings by experts, mark-recapture studies, and other longitudinal studies that do not produce museum voucher specimens, may provide a better representation of their full geographic and climatic ranges. The use of range polygons for habitat modeling has been criticized because some species are known to occur only in specific microhabitats whose climate differs from the surrounding landscapes encompassed in generalized range maps. We recognize the potential downfall of using range occurrences, but our goal is to estimate large-scale geographic changes over long temporal scales rather than to predict precisely where our species occur or do not occur on a small or medium scale landscape. Each of our points sampled at 50 km intervals from a modern geographic distribution has a coarseness that is appropriately similar to the geographic resolution of paleoclimate estimates and fossil assemblages [41], [42]. Map building, intersection, extraction and manipulation were all performed in a combination of ArcGIS, Mathematica 7.0, and MySQL. All further analyses were performed in Mathematica 7.0.

### Climate Envelope Modeling

We chose climate envelope models to describe potential suitable habitat for a species because a method for incorporating climate envelope models with phylogenetic comparative methods has been established [24], [25]. We used 19 climatic variables derived from the 2.5 arc-minutes WorldClim database Version 1.4 to develop climate envelope models [43]. These 19 variables describe means and extremes of temperature and precipitation on a monthly, quarterly, and annual basis and are referred to as bioclimatic variables (Table 1). The bioclimatic variables were sampled at the 50 km point occurrences that represent each species' geographic distribution.

**Table 1:** Nineteen bioclimatic variables derived from the WorldClim database.

Abbreviation	Variable Description
BIO1	Annual Mean Temp (C)
BIO2	Mean Diurnal Range (C)
BIO3	Isothermality (100 * BIO2 / BIO7)
BIO4	Temp Seasonality (100 * SD)
BIO5	Max Temp of Warmest Month (C)
BIO6	Min Temp of Coldest Month (C)
BIO7	Temp Annual Range (C) (BIO5–BIO6)
BIO8	Mean Temp of Wettest Quarter (C)
BIO9	Mean Temp of Driest Quarter (C)
BIO10	Mean Temp of Warmest Quarter (C)
BIO11	Mean Temp of Coldest Quarter (C)
BIO12	Annual Precip (mm)
BIO13	Precip of Wettest Month (mm)
BIO14	Precip of Driest Month (mm)
BIO15	Precip Seasonality (CV)
BIO16	Precip of Wettest Quarter (mm)
BIO17	Precip of Driest Quarter (mm)
BIO18	Precip of Warmest Quarter (mm)
BIO19	Precip of Coldest Quarter (mm)

The specific bioclimatic variables important for each species differ in type and magnitude, so we included all 19 in the climate envelope models. One concern for including too many variables to model a species' suitable habitat is over-fitting the model to the data; however, this is not the case here because the climate data are not being used as independent variables in a statistical model with the aim of explaining variance in the independent data. Rather the variables are used to characterize a multivariate climate space inhabited by the species and the addition of correlated variables does not have the effect of inflating the climate envelope. The bioclimatic variables that are most closely associated with the species' geographic distribution will be the ones that emerge as influential in the suitable habitat models, whereas the ones that are not associated with the geographic distribution will have little or no effect on the suitable habitat models.

We evaluated two variants of a rectilinear climate envelope, BIOCLIM [44], and 5 variants of an ellipsoidal climate envelope, the generalized linear model, GLM [45], to determine which algorithm was the best predictor of the assumed known modern geographic distributions. For BIOCLIM, rectilinear climate envelopes were calculated for each species based on the maxima and minima of points in the 19-dimensional multivariate climate space. In the first variant we used the absolute maximum and minimum along each axis, in the second variant we used the 5^th^ and 95^th^ percentiles to minimize effects of climatic outliers. For the GLM method, a 19-dimensional ellipsoidal climate probability envelope was calculated, in which a parameter is estimated for each climate variable for location and affiliation with other climate variables. This is then transformed into a probability for each geographic point included in the climate envelope using a logit link function. The five variants of the GLM method included points in the distribution of suitable habitat when their p-values were greater than 0.1, 0.2, 0.3, 0.4, and 0.5, respectively. We did not use logistic models or genetic algorithms such as GARP [46] because these approaches are designed to find the best fit of occurrence data to a specific climate to determine where the existing climate is appropriate for the species. Because we are modeling across time, it is expected that correlations between climate variables will change from what they are in the modern climate; rectilinear climate envelopes do not rely on absence data nor do correlations between climate variables affect their boundaries, making the envelope model more appropriate for estimating changing suitable habitat distributions in past climates.

### Quantitative descriptors of distribution models

We used three statistics to describe geographic distributions for the purposes of assessing the fit of suitable habitat model distributions to known distributions and for describing the rate and degree of change in the paleophylogeographic models. The three statistics are: 1) the geographic center, measured in longitude and latitude, and calculated as the mean of each coordinate for all points in the distribution; 2) the standard deviation of points in the distribution relative to the geographic center; and 3) the number of points included in the distribution.

### Selection of the best suitable habitat distribution model

We tested the predictive power of the seven models by comparing their geographic centers, standard deviations, and numbers of points to known species' distributions. We used an independent two-sample t-test, where the geographic center was the point of comparison, the standard deviation served as the variance, and the numbers of points were the degrees of freedom. This method tests the null hypothesis that the modeled distribution is not significantly different from the known one. To test the robustness of the model distributions with respect to the known distributions and to account for non-normality in the spatially distributed dataset, we subsampled known species' distributions down to 25% of the original occurrences and rebuilt the modeled distributions 100 times. To further summarize the fit of each model distribution to the known species' distribution, we calculated an index of overlap (twice the number of shared points by the modeled and known distributions divided by the sum of number of points in both).

### Phylogenetic regression of climate envelopes

**Figure 1:**
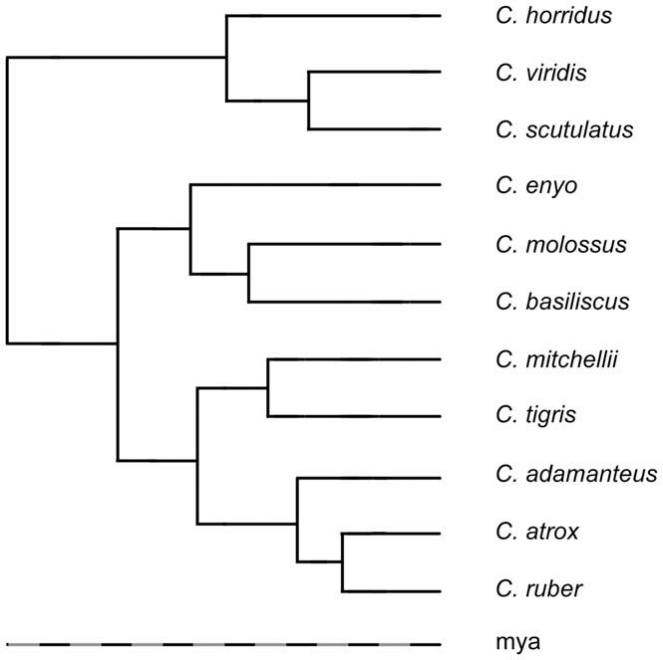
Composite phylogeny of 11 rattlesnakes in the genus *Crotalus*. Phylogenetic relationship of 11 rattlesnake species based on a composite phylogeny from a mixed model Bayesian analysis and maximum parsimony. The color offsets on the bar labeled millions of years ago (mya) are in million year increments.

To construct the evolving climate envelopes, we regressed the climate envelopes of 11 rattlesnake species onto a composite phylogeny [47] based on a mixed model Bayesian analysis [48] and maximum parsimony [49] (Figure 1). Branch lengths were calibrated by averaging divergence times suggested by fossil evidence [36] and genetic distances in molecular phylogeographic studies [37], [38], [39], [40], [50]. We used phylogenetic generalized linear model regression [51] to regress the 95% BIOCLIM envelopes onto the phylogeny using the maximum and minimum of the bioclimatic variables as traits and assuming a Brownian motion model of evolution [23]. This regression yields reconstructions of the most likely envelopes at each of the nodes of the tree. The most likely envelope at any point along a branch of the tree is simply a linear extrapolation between the envelopes at each end of the branch, scaled by the relative position of the point along the branch.

We tested the conformity of the climatic variables with our assumption of a Brownian motion model. The expected absolute divergence of a trait scaled to time since divergence is *D*  =  *rt^a^*, where *D* is the absolute divergence in the trait, *r* is a rate parameter, *t* is the time since divergence, and *a* is a coefficient related to the mode of evolution [52], [53], [54]. The parameter *a* ranges between 0 and 1, where 0 represents stabilizing selection, 0.5 represents perfect Brownian motion (randomly fluctuating selection or genetic drift), and 1 represents perfect diversifying selection. We used maximum likelihood to estimate *r* and *a* for each trait from the pairwise differences between the values at two tips relative to the interval of phylogenetic time connecting the two tips on the tree [53].

### Interpolated paleoclimate maps

We used modern climate [43] and the GCM MICROC3.2 model of the LGM (∼21 kya) from PMIP2 [31] to interpolate climates between and beyond these data. We tested our interpolated estimates of paleoclimate against independent GCMs for two times, 6 kya and 120 kya. We estimated mean annual temperature and annual precipitation by interpolating proportionally between our two end-member climate datasets, scaling the interpolation by composite benthic stable oxygen isotope ratios [33]. The stable oxygen isotope ratios are derived from multiple cores at a site in the North Atlantic Ocean on the western flank of the Mid-Atlantic Ridge. They are a proxy for northern hemisphere to global temperature and probably have less noise than terrestrial cores because they are deposited in a more stable environment.

We chose to test the climate interpolation at 6 kya because several GCMs have been used to model climate then (notably CCSM and MICROC3.2), allowing us to determine whether our interpolated model is as similar to the GCMs as they are to each other. We also compared the differences between a GCM from the last interglacial [32] with our climate interpolation model. Because our purpose is to facilitate the study of the continental-scale response of terrestrial animal habitats to climate change, we restricted our test to North America and used 50 km points as our level of resolution.

### Construction of paleophylogeographic models and their relationship with change in temperature

**Figure 2:**
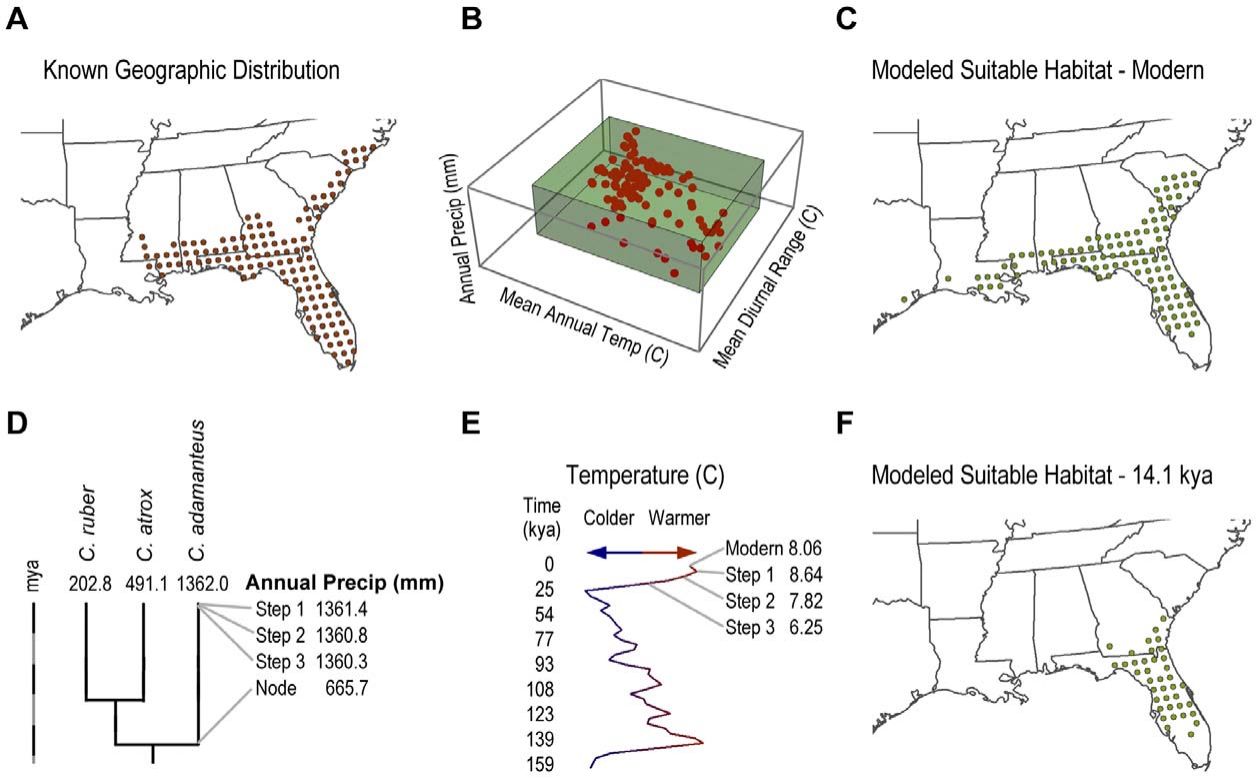
Illustration of a paleophylogeographic model. A, Modern geographic distribution for *Crotalus adamanteus*. B, Climate envelope for three bioclimatic variables. The red points represent the climate associated today with each red 50 km point from the modern geographic distribution in A. The green cube represents the climate envelope, the 5^th^ and 95^th^ percentile of each of the three bioclimatic variables. C, Suitable habitat modeled from the climate envelope on the modern climate. The green 50 km points on the map all fall within the green climate space in B and are considered suitable habitat for *C. adamanteus* today. D, Phylogeny and ancestral reconstructions. Annual Precipitation (mm) is regressed on the phylogeny. The first three steps of the reconstruction are shown at 4.7 kya, 9.4 kya and 14.1 kya. E, Temperature estimates for the North American continent derived from a composite oxygen isotope curve. The paleoclimate reconstruction for each step is scaled based on this curve. F, Phylogenetically scaled climate envelope projected onto isotopically scaled paleoclimate model at 14.1 kya. These 50 km points are considered suitable habitat for *C. adamanteus* 14.1 kya.

Paleophylogeographic models were reconstructed in increments through the last 320 ky by projecting the phylogenetically-scaled climate envelopes onto concurrent paleoclimate maps (Figure 2). Increments were approximately 4 ky, but specifically determined by age estimates of oxygen isotope values [33]. We calculated the average change in geographic center (km) and areal extent (km^2^) for all incremented time steps that included only phylogenetically scaled climate envelopes, only paleoclimate models, and both. *Crotalus enyo*, *C. adamanteus*, and *C. ruber* were excluded because of their intermittently undetectable suitable habitat at a 50 km scale. Because these species are modeled to have lost their entire suitable habitat at a 50 km scale during extreme climate, they are probably the most vulnerable to excessive climate change and excluding them from our further analyses provides us with a highly conservative estimate of geographic shifts due to expected evolutionary change or climate change. All pairwise comparisons of average change in geographic center and areal extent were linearly regressed on corresponding change in mean annual temperature (°C) with a bootstrapped estimate of standard error and a randomization to account for non-normally distributed data.

### Verification with the fossil record

To assess the validity of the paleophylogeographic models we compared the predicted past distribution of *Crotalus* to known occurrences in the Pleistocene fossil record by testing the overlap of past modeled distributions and fossil occurrences. Forty-one documented occurrences [36] were downloaded from the Paleobiology Database on 11 May, 2011, using the genus name ‘*Crotalus*’. Species level identification is not commonly reported for these rattlesnakes in the Paleobiology Database, so the comparison was generalized to the genus level. The clade of rattlesnakes that we modeled only represent about one third of the recognized species in this genus, so our sample does not represent the niche diversity of the genus. However, several of the species within this clade represent the northern extent of the current known distribution of the genus. Although the species' suitable habitat at the northern extent of the distribution of the genus does not necessarily represent the harshest climates (e.g., montane species in Mexico live in more extreme climates), they do represent the northern extent of the genus geographically. Much of the North American continent is covered by different species within this genus, so paleodistribution models of the northern extent of rattlesnake suitable habitat provides a testable hypothesis. If a fossil occurs north of the northernmost edge of the modeled suitable habitat, it is clear that the models are incorrect.

### Projection of climate envelopes on future climate scenarios

Suitable habitats were modeled under two future climate scenarios for the year 2100 for 11 rattlesnake species. The two climate scenarios were developed for an increase in mean annual temperature by 1.1°C and by 6.4°C, the range of possible mean annual temperature increases reported by the IPCC [1]. Future climate maps were constructed by the climate interpolation method described above in ‘Interpolated paleoclimate maps’ using the change in stable oxygen isotope values as a proxy for change in temperature.

## Results

### Selection of the best climate envelope model

BIOCLIM envelope models fared better than GLM envelope models to quantitatively characterize the 11 rattlesnake species' known distributions, where all GLM models with varying parameters were statistically distinguishable from known distributions (Table S1 and S2). The 95% BIOCLIM model fared best (8 of the 11 modeled habitat distributions were statistically indistinguishable from the documented modern species' geographic distribution) and we used it for the remaining analyses (Table S1 and S2).

### Evolutionary mode of climate variables

All climate variables considered in our climate envelope models either evolved under a Brownian motion model of evolution or under stabilizing selection (Table 2). In either case, a Brownian motion model of evolution is adequate to model the most likely ancestral nodes across a tree and was used to phylogenetically regress our climate envelope models.

**Table 2:** Maximum likelihood estimate of evolutionary rate and mode.

Bioclimate Variable	*r*	P	*α*	P	Evo Mode
Annual Mean Temp	10.60	0.17	0.66	0.21	Random
Mean Diurnal Range	20.15	0.46	0	1	Stabilizing
Isothermality	3.69	0.58	0.39	0.70	Random
Temp Seasonality	805.38	0.83	0.41	0.49	Random
Max Temp of Warmest Month	7.57	0.38	0.51	0.71	Random
Min Temp of Coldest Month	20.13	0.76	0.55	0.08	Random
Temp Annual Range	26.69	0.95	0.33	0.46	Random
Mean Temp of Wettest Quarter	23.09	0.90	0.39	0.29	Random
Mean Temp of Driest Quarter	20.74	0.33	0.47	0.63	Random
Mean Temp of Warmest Quarter	6.79	0.20	0.65	0.24	Random
Mean Temp of Coldest Quarter	18.07	0.78	0.60	0.70	Random
Annual Precip	579.12	0.99	0	0.99	Stabilizing
Precip of Wettest Month	86.99	0.27	0	1	Stabilizing
Precip of Driest Month	30.57	0.47	0.05	1	Stabilizing
Precip Seasonality	17.27	0.29	0.29	0.99	Stabilizing
Precip of Wettest Quarter	240.34	0.15	0	1	Stabilizing
Precip of Driest Quarter	116.99	0.61	0.02	1	Stabilizing
Precip of Warmest Quarter	208.06	0.60	0	1	Stabilizing
Precip of Coldest Quarter	123.14	0.28	0	1	Stabilizing

Maximum likelihood estimate of evolutionary rate, *r*, a coefficient related to the mode of evolution, *α*, and the corresponding evolutionary mode (Evo Mode). Evolutionary mode is characterized as either stabilizing (stabilizing selection), random (randomly fluctuating selection or genetic drift), or diversifying (diversifying selection). P values are derived from 10,000 permutations of the maximum likelihood estimate.

### Paleoclimate interpolation

**Figure 3:**
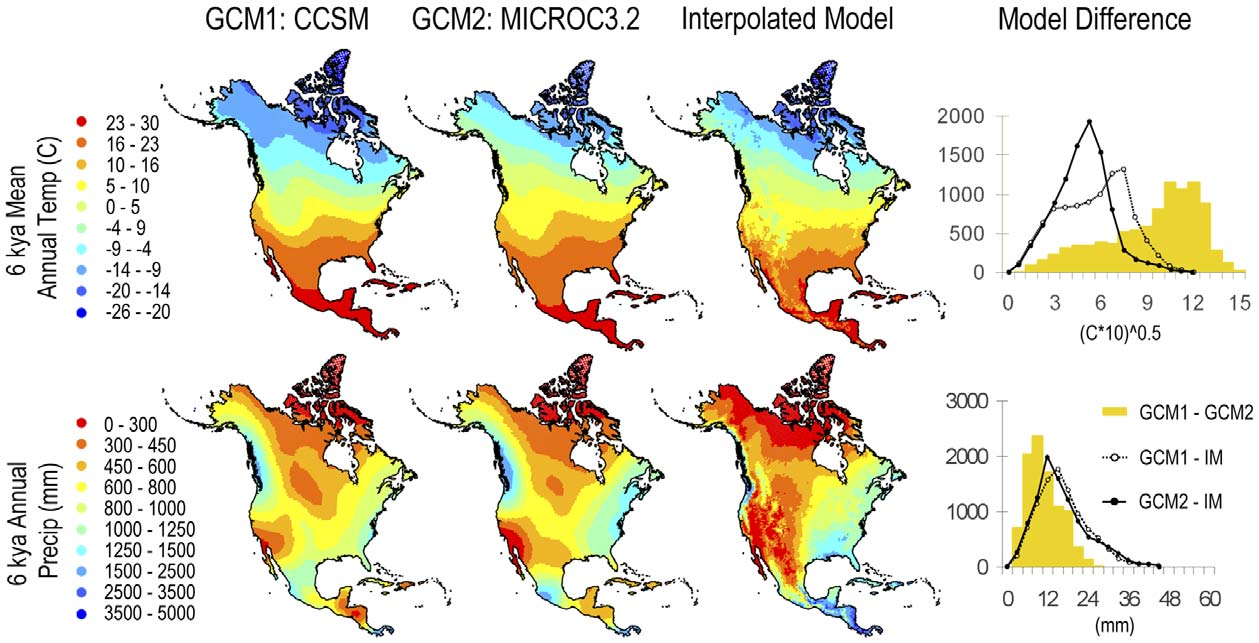
Mean annual temperature and annual precipitation modeled for 6 kya. Two general circulation models (GCM1 and 2) and our interpolation model (IM) are compared. Graphs at the right show histograms of the absolute differences between the two GCMs (yellow bars) and between our model and each of the GCMs (lines).

**Figure 4:**
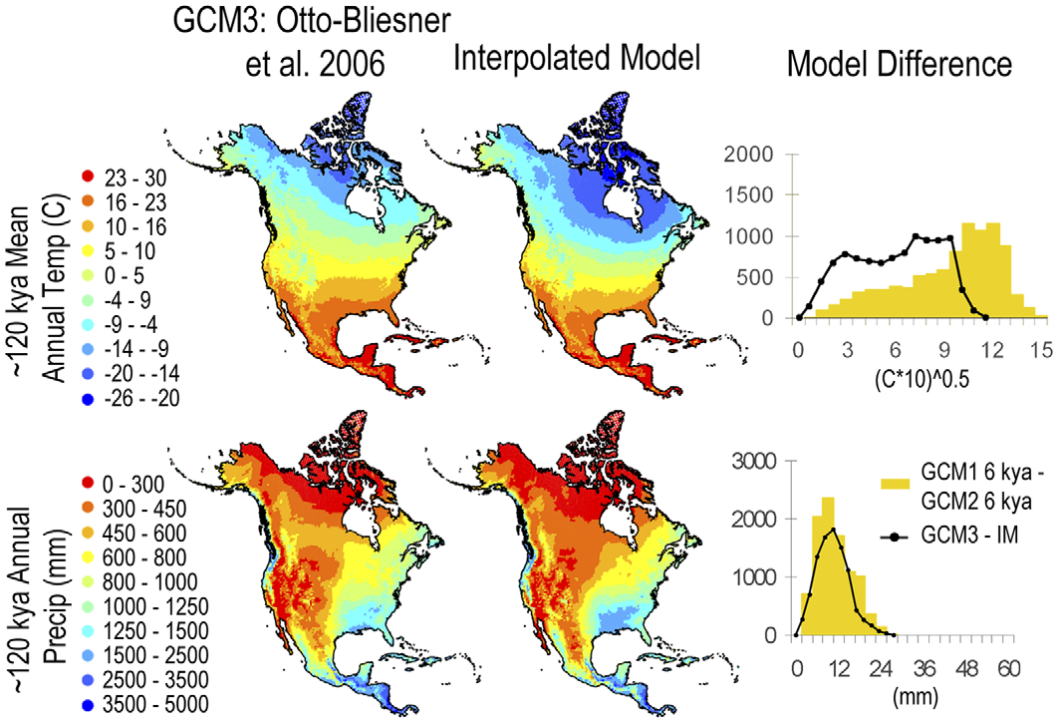
Mean annual temperature and annual precipitation modeled for ∼120 kya. One GCM and an interpolation model (IM) are compared. Graphs at the right show differences between our model and the GCMs (line) with the differences between the two 6 kya GCMs for comparison (yellow bars).

Our interpolated model of mean annual temperature at 6 kya was consistent with the corresponding GCMs; in that, it was no more different from either GCM as the two GCMs were from each other (Figure 3). Note here that we are testing if the difference distribution between the interpolated model and either climate model is greater than the difference distribution between the two GCMs, not if the distributions match. That our interpolated model of mean annual temperature was accurate (the differences between the interpolated model and either GCM are less than or equal to the difference between the two GCMs) is no surprise since the stable isotope values used to make the interpolation are themselves a proxy of mean annual temperature, albeit one that does not contain information about geographic variation of temperature across the continent. More importantly, our interpolated model of annual precipitation, which is derived entirely from the spatial correlation between precipitation and temperature in our two end-member climate models and our temperature-based interpolation between them, also compared favorably with the two GCMs (Figure 3). Furthermore, our climate interpolations for the last interglacial, which lies outside our two end-member models, were as similar to the last interglacial GCM as our 6 kya interpolations were to their corresponding GCMs (Figure 4).

### Paleophylogeographic models through time

**Figure 5:**
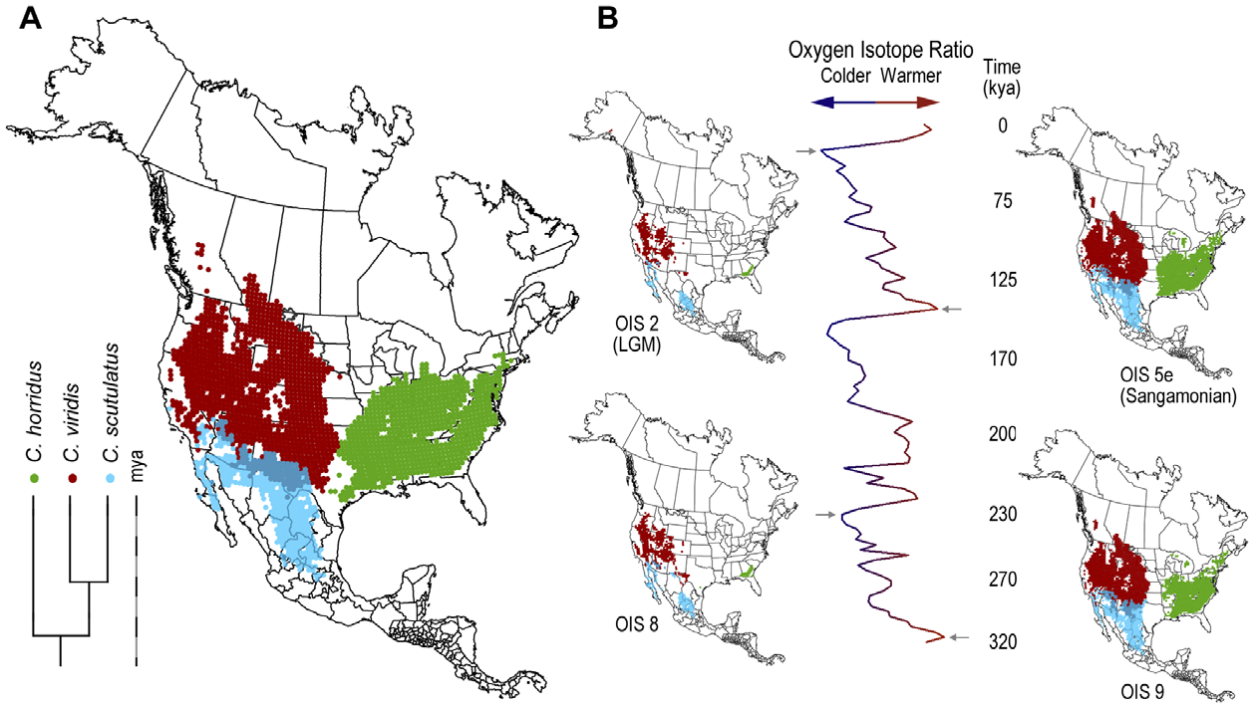
Paleophylogeographic distribution models for three species of rattlesnake (*Crotalus*). A, Phylogeny and modern geographic distribution models mapped onto modern climatic conditions. The dark gray curve represents the southern extent of glaciers during the LGM. B, Composite oxygen isotope curve for the last 320 ky inset with four paleophylogeographic reconstructions at four points, two glacial and two interglacial, to illustrate the effects of climate changes and phylogeny on the distribution of suitable habitats. Phylogenetically scaled climate envelopes were projected onto isotopically scaled paleoclimate models to generate these maps. Supplemental videos show animations of the paleophylogeographic distributions through the last 320 ky for these three species (Video S1) and for the remaining species (Video S2 and S3).

Figure 5 shows how the paleophylogeographic distribution of suitable habitats of three rattlesnakes is expected to have changed through time. The potential for dramatic changes in the location and areal extent of suitable habitat are particularly apparent in the paleophylogeographic models for glacial times (Figure 5B). Among the eleven rattlesnakes species studied here, suitable habitats have expanded rapidly northward since the LGM in *Crotalus adamanteus*, *C. enyo*, *C. horridus*, and *C. tigris* (see Video S1, S2, and S3). At some times in the past, suitable habitat for a few species (*C. adamanteus*, *C. enyo*, and *C. basiliscus*) was so constricted that it was undetectable at the 50 km resolution of our models.

### Fossil Record Occurrences

**Figure 6:**
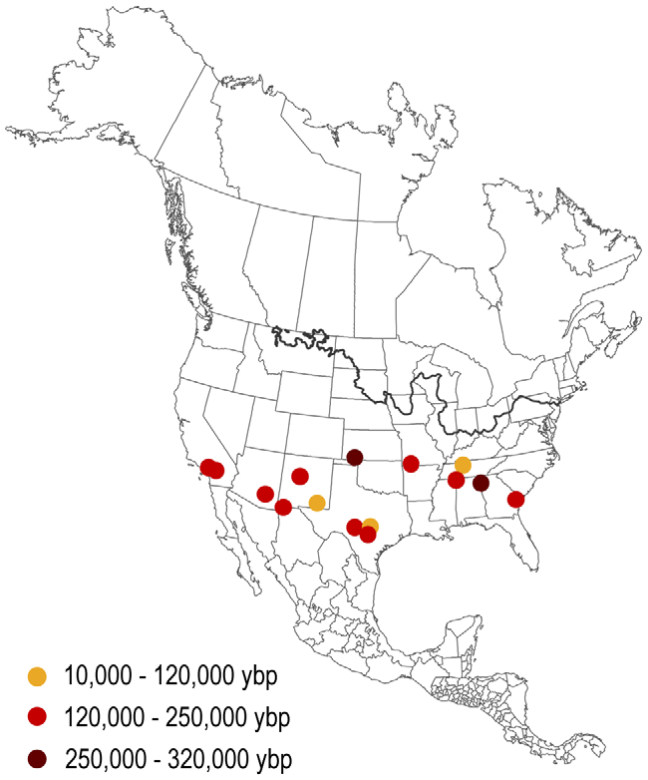
Fossil occurrences of *Crotalus* in North America for the last 320,000 years. Orange points show occurrence sites whose maximum ages are less than 120 kya, red points show sites with maximum ages between 120–250 kya, and brown points show sites with maximum ages between 250–320 kya. The data were downloaded from the Paleobiology Database (http://pbdb.org) on 22 May 2011, using the group name ‘*Crotalus*’.

All described occurrences of *Crotalus* from the Quaternary fossil record are consistent with the predicted range of our paleophylogeographic models as far as temporal control on the fossil sites permits comparisons (Figure 6). However, we note that the fossil record of these snakes is poor and does not provide a powerful test of the details of our models, highlighting the need for further study of these paleoclimatically informative animals.

### Paleophylogeographic models and their relationship to temperature change

**Figure 7:**
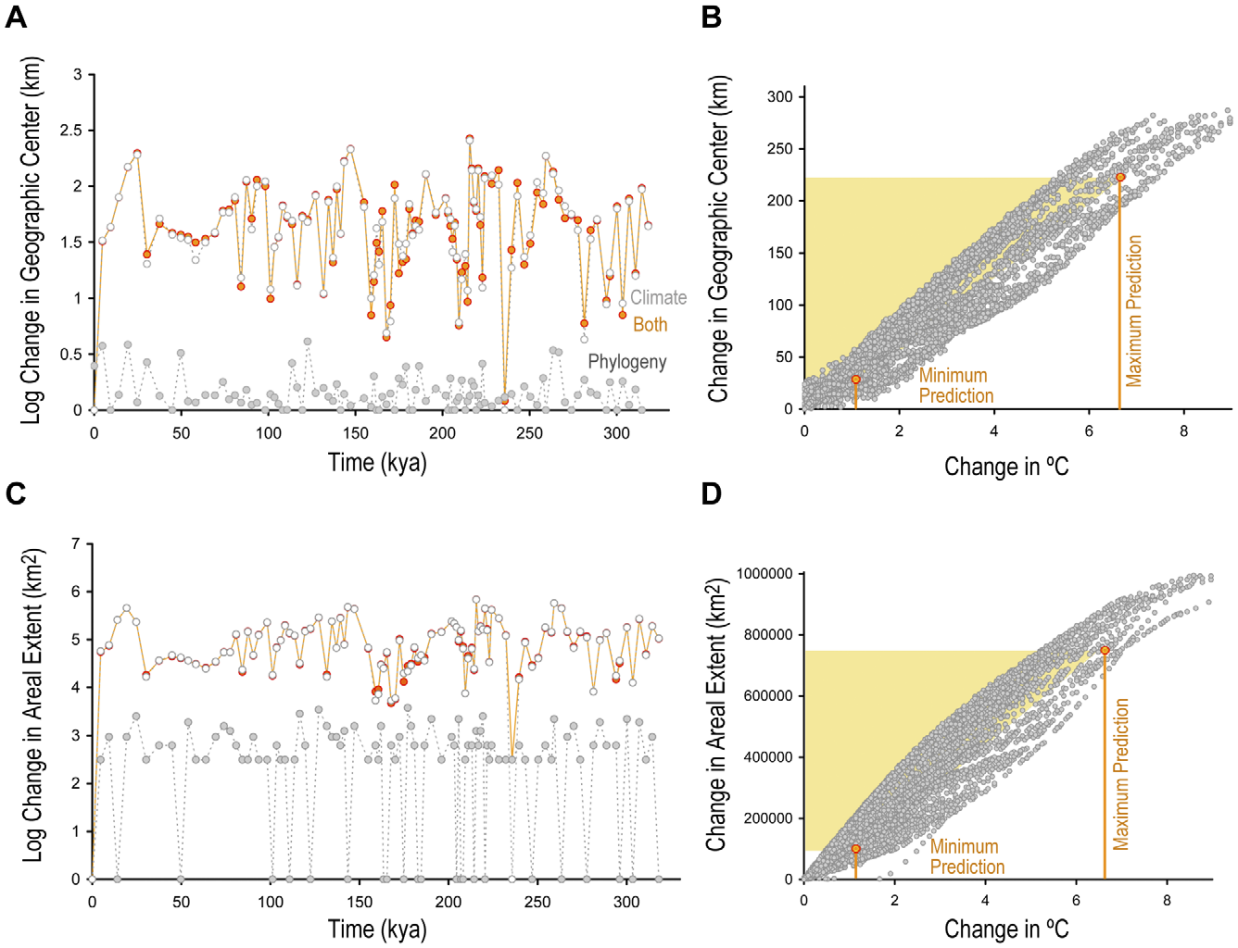
Average change in species' distributions of suitable habitat over the last 320 ky. A, Time series of change in geographic center (km). Change due to climate and phylogeny are modeled separately to identify the contributions of each in a model incorporating change due to both climate and phylogeny. B, Change in geographic center as it relates to temperature (°C). C, Time series of change in areal extent (km^2^). D, Change in areal extent as it relates to temperature change (°C). The shaded area indicates increase in global temperature by the end of the 21^st^ century predicted by IPCC 2007.

Climate has contributed more to changes in modeled suitable habitat of these rattlesnakes than evolutionary change by two to three orders of magnitude over the last 320 ky (Figure 7). The historical change in mean annual temperature has been strongly correlated with both change in geographic center (R^2^ = 0.915±0.002 s.e.; *p*<0.0001; *n* = 5,050) and areal extent (R^2^ = 0.924±0.002 s.e.; *p*<0.0001; *n* = 5,050) of these species' suitable habitats (Figures 7B and 7D). Based on this correlation, we estimate that the centers of these habitats have on average been displaced by 34.93 km per°C and their areal extents have changed by 121,591 km^2^ per°C. A pairwise comparison of average changes between time intervals (*n* = 5,050) reveals a 0.0023 km/year average rate of displacement.

### Projection of climate envelopes on future climate scenarios

**Figure 8:**
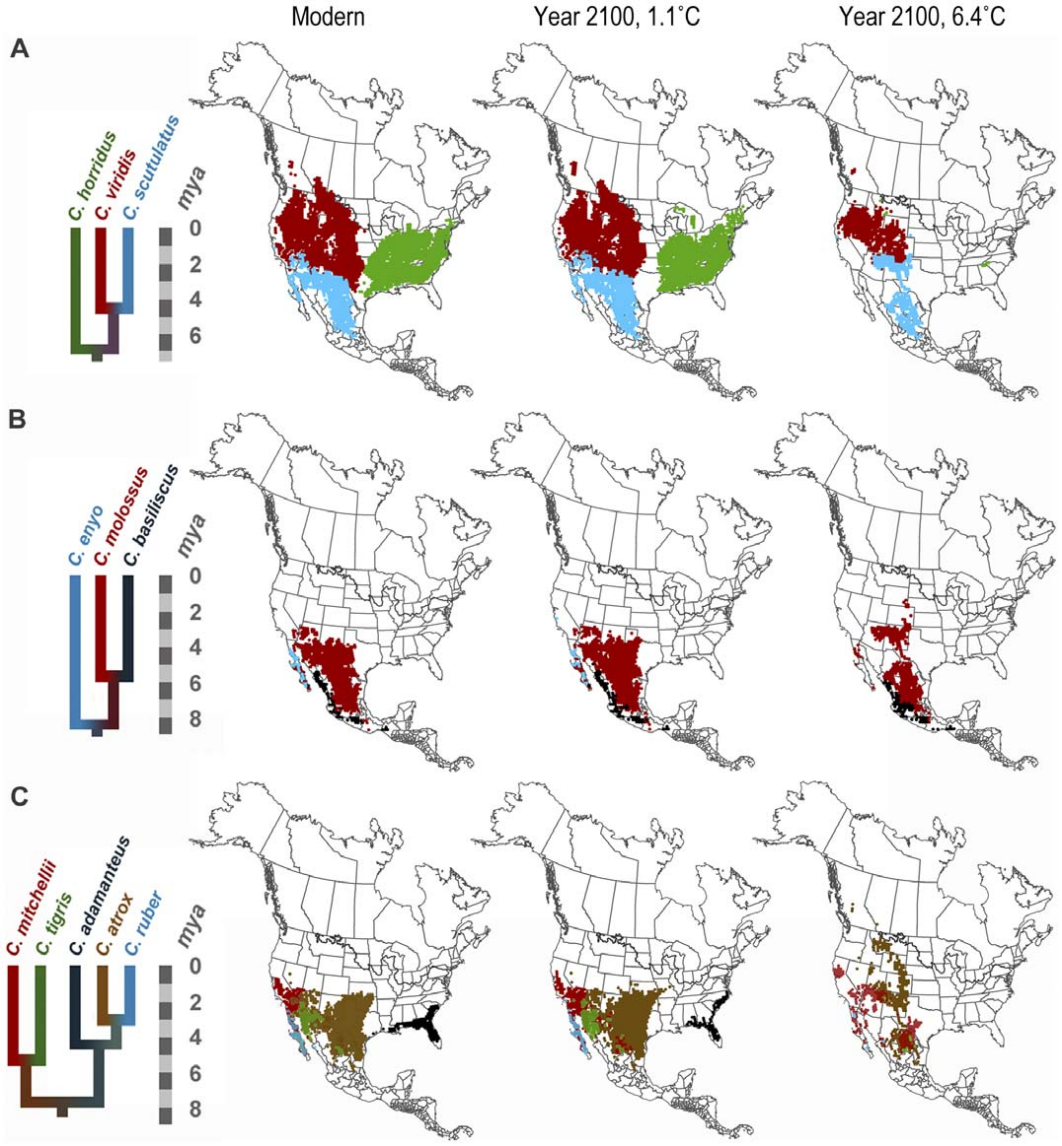
Current and future predictions of suitable habitat. Suitable habitat distributions were modeled under two future climate scenarios for the year 2100 for 11 rattlesnake species. The two climate scenarios are derived from an increase of mean annual temperature by 1.1°C and by 6.4°C. A, B, and C, Phylogeny and modeled suitable habitat distributions by clade.

If we extrapolate from our estimates of Pleistocene rates of change per°C, the average displacement of the centers of species' suitable habitats in the next 90 years will be 38.31–217.49 km (0.43–2.42 km/yr) and their areal extents will change by 133,783–799,963 km^2^ (Figure 8). The rates of current displacement are two to three orders of magnitude greater than the rate of change we measured through the dramatic climatic fluctuations of the last 320 ky, 0.0023 km/year.

## Discussion

Reasonable multi-parameter spatial estimates of past Quaternary climates can be produced by interpolation from end-member GCMs using a proxy for a single climate parameter (mean annual temperature), provided that the end-members represent the extremes of the paleoclimates that are being estimated. Despite good agreement of our interpolated paleoclimate models with the GCM of the last interglacial, it is likely that our method will produce increasingly inaccurate models the further they are extrapolated from the end-member climate data. In addition, interpolated paleoclimate models would have an increased accuracy with the inclusion stable oxygen isotope geographic variation. This computationally straightforward tool does not replace the need for GCMs, but it facilitates the modeling of dynamic changes in past climates at a continental scale, which is especially useful for studies of species' responses to past climate change.

During the last 320 ky, three major glacial cycles have come and gone, the global mean annual surface temperature has varied by 6 to 8°C, and some of the warmest and coldest global temperatures of the last million years have occurred, forcing species to repeatedly shift either their geographic distributions or their climatic tolerances. We suggest that physiological limits or climatic tolerances did not shift rapidly, because of the phylogenetic component of this analysis. The variance in the bioclimatic variables across the entire clade does not necessarily encompass the extreme variations of a fixed 50 km point through the glacial-interglacial cycles (Table 3). For example, the annual mean temperature in Bloomington, IN is 11.5°C. Several of the rattlesnakes encompass that temperature as part of the range of the annual mean temperatures that they currently inhabit. However, the annual mean temperature during the LGM in Bloomington, IN was −8.5°C and none of the species come close to including that temperature within their range of annual mean temperatures. The same is true for many of the bioclimatic variables and in Table 3 we show these ranges for annual mean temperature, minimum temperature of the coldest month, maximum temperature of the hottest month, and temperature seasonality.

**Table 3:** Example of climate fluctuations for Bloomington, IN and species' ranges.

	Annual Mean Temp	Min Temp of Coldest Month	Max Temp of Warmest Month	Temp Seasonality
**Bloomington, IN**				
Present	11.5	−7.3	29.9	950
LGM	−8.5	−29.1	11.6	910
120 kya	9.7	−22.8	38.4	1408
**Species**' **Ranges**				
*C. horridus*	2.5 – 21.5	−17.9 – 6.9	21.0 – 35.9	562 – 1212
*C. viridis*	−3.8 – 22.1	−22.6 – 5.6	15.7 – 38.0	543 – 1245
*C. scutulatus*	5.9 – 24.2	−9.6 – 14.7	14.9 – 43.2	106 – 949
*C. enyo*	15.2 – 23.9	3.4 – 12.3	25.8 – 37.1	336 – 502
*C. molossus*	5.9 – 28.2	−11.0 – 16.5	14.9 – 41.4	106 – 864
*C. basiliscus*	15.0 – 28.8	3.1 – 17.7	24.6 – 39.9	141 – 512
*C. mitchellii*	7.5 – 24.3	−6.7 – 12.3	25.4 – 42.2	336 – 913
*C. tigris*	15.0 – 25.7	−1.7 – 11.3	31.7 – 41.2	512 – 827
*C. adamanteus*	15.9 – 23.8	−0.8 – 13.7	31.5 – 33.8	332 – 776
*C. atrox*	6.7 – 27.6	−10.6 – 19.6	23.3 – 42.2	145 – 974
*C. ruber*	10.1 – 23.9	−3.6 – 12.3	25.8 – 38.9	336 – 740

Example of the variance of 4 bioclimatic variables for one 50 km point in the Midwest, Global ID #139912, (Bloomington, IN) and the range of these variables in the modern climate envelopes for 11 rattlesnake species.

Large fluctuations in climate from the glacial-interglacial cycles did not erase the phylogenetic signal present in bioclimatic variables describing species' climate envelopes. We tested the assumption of a Brownian motion-like model of evolution for the phylogenetic regression of bioclimatic variables (Table 2). The mean diurnal range and all variables related to precipitation have an evolutionary mode of stabilizing selection, suggestive of phylogenetic niche conservatism [55]. The remaining temperature variables have between species variation that is consistent with a Brownian motion-like model of evolution, suggestive of phylogenetic signal and not conservatism. In either case, diversifying or divergent selection was not identified for any bioclimatic variable describing the climate envelopes, and ancestral climate envelopes could be reconstructed.

The rate at which climate has forced change in suitable habitat in the rattlesnakes we studied has been much more rapid than changes in climate envelopes associated with macroevolution or speciation (Figure 7). However, our paleophylogeographic models do not incorporate anagenesis apart from a gradual linear change since speciation; therefore, the phyletic evolution modeled is dampened by modeling an average trait value for the climate envelopes through time. The phylogeny we use to model phyletic evolution has roughly estimated divergence dates attached to it and we do not incorporate a range of possible divergence time estimates in our phylogenetic regression. It is likely that our calculations of the change in available suitable habitat will minimally change if we incorporated ranges in divergence times. We estimate ancestor nodes for divergences that happened millions of years ago, so the extrapolation to the last 320 ky will only minutely change the effects of phylogeny from the estimated ancestor.

In this same regard, the results in Figure 7 are expected because phylogenetic change is modeled with a constant rate since the spilt of the last common ancestor millions of years ago. So the modeled change on 4 ky time increments eventually produces the larger shifts that we see between species' climatic envelopes. Although we would expect this outcome for this particular group of species, due to the deeper divergences, it is not necessarily known what to expect for other species groups. Our intention here is to develop an interdisciplinary framework that can be extended to deeper time contexts and for other species groups.

The amount of evolutionary change is probably underestimated in our paleophylogeographic models, because adaptive changes that accumulate within glacial or interglacial periods may effectively be erased by the cycling climate and not incorporated in macroevolutionary change [56]. Accounting for potential phyletic evolution increases model complexity and is beyond the scope of this study. Future investigations should incorporate a stochastic parameter to model within species variation per generation, because evolutionary dynamics can happen over even short time scales [57], [58]. In addition, future study might investigate the evolution of dispersal ability, which potentially has a large effect on the geographic limits of a species' distribution [59]. Regardless, the velocity with which climate is now changing will shift entire biomes rapidly and individual species will have to shift geographically at very high rates to keep up [60].

The future projected displacements in rattlesnake suitable habitat we calculated of 0.43–2.42 km/yr are similar to those observed over recent decades in other species (0.61 km/yr) [7]. Nevertheless, these projected rates are two to three orders of magnitude greater than the expected or background rate of displacement in rattlesnakes (0.0023 km/yr) that we modeled through the dramatic climate fluctuations of the last 320 ky. Those species whose suitable habitat changed the most markedly through the Pleistocene climate cycles may be ones that are particularly sensitive to current climatic fluctuations and habitat alterations. Our paleophylogeographic models suggest that *C. adamanteus*, the Eastern diamondback rattlesnake, is one of these particularly sensitive species. Conservation studies have already documented a decline in this species' abundance in association with habitat destruction and fragmentation [61], [62].

Based on the rate in the fastest changing species (*C. horridus*), future displacement of the center of a species' suitable habitat could be as high as 144.91–711.32 km (1.61–7.90 km/yr) and a change in areal extent as much as 257,243–1,526,050 km^2^. Geographic displacements of this magnitude are many times larger than the size of current natural preserves; for example, the largest US National Park, Wrangell-St. Elias Preserve in Alaska, is only 53,418 km^2^. Although suitable habitat may increase (or decrease) in areal extent as global temperature increases, the location of that habitat may be displaced faster or farther than a species is able to track, and may result in a population bottleneck that could lead to extinction.

Every species has its own particular relation to climate and environment, of course, but our results are generalizable beyond rattlesnakes. Any species that has a similar sized geographic distribution today at similar latitudes will have experienced the same climatic changes in the past and will experience the same ones in the near future. We predict that the rapidity and magnitude of changes in other terrestrial vertebrates will be similar, on average, to what we found in rattlesnakes, a prediction that is substantiated by the similar rates of geographic expansion that have been observed in other species in recent decades (e.g., [7], but see [63]).

The modeling of species' distributions through time illustrates how dramatically they can contract during glacial periods, expand in interglacial periods, and fragment and coalesce in complicated ways in between. The ability of these 11 rattlesnake species to persist through the last 320 ky, when suitable habitat was repeatedly fragmented and reorganized, suggests that conservation strategies that create habitat corridors [64] or rely on managed relocations may be successful [60], [65]. The ways in which species responded to past climatic shifts provides a framework for how we expect species to respond in the future. Knowledge about the past allows for more informed conservation decisions in the present, which are vital for the preservation of species' biodiversity in our rapidly changing global climate.

## Supporting Information

Table S1 **Quantitative descriptors of known geographic distributions and climate envelope models for 11 rattlesnake species.** Standard errors were estimated by recalculating each statistic from 100 random subsamples of 90% of the points in each known distribution. Geographic center (GC) is the centroid of the geographic distributions reported in latitude and longitude, WGS 1984. Areal extent is the number of 50 km points that the geographic distribution covers.
(DOC)

Table S2 **Tests for the fit and stability of climate envelope models.** Independent two sample t-tests for departure of modeled distributions from the known species' distribution (Model Fit) and for departure of 100 randomly subsampled models, each at 25% of the original data, from the known species' distribution (Model Stability). For model fit, the P values indicate the probability that the model is identical to the known distribution apart from chance. An asterisk indicates the model is significantly different from the known distribution. For model stability, the P value indicates the mean probability that the subsampled models are identical to the climate envelope model except by chance. Overlapping proportion (OP) is the proportion of points in the model and the known distribution.
(DOC)

Video S1 **Paleophylogeographic model for three rattlesnake species (** ***Crotalus horridus*** **,** ***C. viridis*** **, and** ***C. scutulatus*** **).** A, Phylogeny and suitable habitat models mapped onto climatic conditions from corresponding time intervals to illustrate the effects of climate and phylogenetic changes on the distribution of suitable habitats. Phylogenetically scaled climate envelopes were projected onto isotopically scaled paleoclimate models to generate these maps. The dark gray curve represents the southern extent of glaciers during the LGM. B, Composite oxygen isotope curve for the last 320 ky inset with a yellow circle to indicate the oxygen isotope ratio and the time interval used in the model on the adjacent map.
(GIF)

Video S2 **Paleophylogeographic model for three rattlesnake species (** ***Crotalus enyo*** **,** ***C. molossus*** **, and** ***C. basiliscus*** **).** A, Phylogeny and suitable habitat models mapped onto climatic conditions from corresponding time intervals to illustrate the effects of climate and phylogenetic changes on the distribution of suitable habitats. Phylogenetically scaled climate envelopes were projected onto isotopically scaled paleoclimate models to generate these maps. The dark gray curve represents the southern extent of glaciers during the LGM. B, Composite oxygen isotope curve for the last 320 ky inset with a yellow circle to indicate the oxygen isotope ratio and the time interval used in the model on the adjacent map.
(GIF)

Video S3 **Paleophylogeographic model for five rattlesnake species (** ***Crotalus mitchellii*** **,** ***C. tigris*** **,** ***C. adamanteus*** **,** ***C. atrox*** **, and** ***C. ruber*** **).** A, Phylogeny and suitable habitat models mapped onto climatic conditions from corresponding time intervals to illustrate the effects of climate and phylogenetic changes on the distribution of suitable habitats. Phylogenetically scaled climate envelopes were projected onto isotopically scaled paleoclimate models to generate these maps. The dark gray curve represents the southern extent of glaciers during the LGM. B, Composite oxygen isotope curve for the last 320 ky inset with a yellow circle to indicate the oxygen isotope ratio and the time interval used in the model on the adjacent map.
(GIF)
