# Supplementary material for: Pleistocene Climate, Phylogeny, and Climate Envelope Models: An Integrative Approach to Better Understand Species' Response to Climate Change
Source: PLoS One. 2011 Dec 2;6(12):e28554. doi: 10.1371/journal.pone.0028554 (PMC3229599; doi:10.1371/journal.pone.0028554)
Supplement: Table S1 — Quantitative descriptors of known geographic distributions and climate envelope models for 11 rattlesnake species. Standard errors were estimated by recalculating each statistic from 100 random subsamples of 90% of the points in each known distribution. Geographic center (GC) is the centroid of the geographic distributions reported in latitude and longitude, WGS 1984. Areal extent is the number of 50 km points that the geographic distribution covers. (DOC) [file pone.0028554.s001.doc]

**Table S1.**

|  | GC | Std Err (km) | Std Dev (km) | Areal Extent |
| --- | --- | --- | --- | --- |
| **Known Distribution** | |  |  |  |
| *C. horridus* | 36.0363, -86.7796 | 7.07 | 710.32 ± 3.40 | 873 ± 18.80 |
| *C. viridis* | 40.1736, -110.475 | 6.93 | 814.80 ± 2.45 | 1361 ± 23.70 |
| *C. scutulatus* | 29.0531, -106.58 | 11.38 | 706.14 ± 6.80 | 347 ± 13.50 |
| *C. enyo* | 27.8435, -113.396 | 39.11 | 350.19 ± 29.45 | 13 ± 10.20 |
| *C. molossus* | 28.1085, -105.468 | 9.33 | 684.35 ± 4.45 | 565 ± 15.70 |
| *C. basiliscus* | 22.8416, -105.936 | 17.68 | 357.90 ± 7.44 | 48 ± 10.50 |
| *C. mitchellii* | 31.6878, -114.274 | 10.22 | 429.59 ± 7.68 | 109 ± 11.10 |
| *C. tigris* | 30.7837, -111.195 | 13.51 | 234.26 ± 7.76 | 31 ± 10.40 |
| *C. adamanteus* | 30.4118, -83.3751 | 10.74 | 383.01 ± 5.58 | 123 ± 11.30 |
| *C. atrox* | 30.2222, -103.724 | 7.57 | 666.01 ± 3.63 | 766 ± 17.70 |
| *C. ruber* | 27.4943, -113.146 | 20.93 | 400.38 ± 15.27 | 37 ± 10.40 |
| **BIOCLIM (Min-Max)** | |  |  |  |
| *C. horridus* | 37.5941, -86.2653 | 5.37 | 779.99 ± 3.80 | 1275 ± 14.45 |
| *C. viridis* | 40.9421, -108.966 | 16.47 | 1236.55 ± 18.14 | 2405 ± 40.42 |
| *C. scutulatus* | 29.4096, -107.046 | 26.00 | 852.93 ± 13.68 | 804 ± 36.10 |
| *C. enyo* | 27.3336, -113.164 | 26.37 | 286.92 ± 24.81 | 25 ± 4.30 |
| *C. molossus* | 28.8939, -105.013 | 30.16 | 879.86 ± 53.16 | 976 ± 35.78 |
| *C. basiliscus* | 21.8778, -104.362 | 29.28 | 472.66 ± 18.52 | 81 ± 5.61 |
| *C. mitchellii* | 31.494, -111.504 | 22.91 | 710.76 ± 25.59 | 350 ± 16.37 |
| *C. tigris* | 30.4657, -110.338 | 28.86 | 335.11 ± 43.61 | 75 ± 8.36 |
| *C. adamanteus* | 30.7222, -84.2478 | 7.44 | 438.46 ± 6.946 | 163 ± 5.19 |
| *C. atrox* | 30.8974, -101.008 | 38.37 | 1193.01 ± 30.98 | 1814 ± 88.36 |
| *C. ruber* | 27.612, -112.808 | 8.64 | 412.01 ± 29.30 | 50 ± 3.94 |
| **BIOCLIM (5%-95%)** | |  |  |  |
| *C. horridus* | 36.2214, -87.0283 | 3.56 | 638.13 ± 1.30 | 769 ± 5.71 |
| *C. viridis* | 39.9545, -109.615 | 3.92 | 790.71 ± 2.70 | 1169 ± 10.82 |
| *C. scutulatus* | 28.8723, -106.418 | 7.17 | 597.25 ± 6.00 | 393 ± 10.12 |
| *C. enyo* | 27.8478, -113.441 | 40.98 | 252.18 ± 34.42 | 24 ± 5.11 |
| *C. molossus* | 28.3166, -105.551 | 12.60 | 620.36 ± 9.51 | 531 ± 10.23 |
| *C. basiliscus* | 21.5472, -103.691 | 23.62 | 555.23 ± 20.03 | 72 ± 3.45 |
| *C. mitchellii* | 31.9058, -113.474 | 17.90 | 570.24 ± 15.16 | 191 ± 9.20 |
| *C. tigris* | 30.7171, -110.833 | 32.05 | 360.34 ± 32.18 | 76 ± 5.83 |
| *C. adamanteus* | 30.529, -83.95 | 19.01 | 373.93 ± 16.47 | 106 ± 7.37 |
| *C. atrox* | 31.1672, -104.215 | 8.54 | 620.32 ± 5.45 | 654 ± 9.57 |
| *C. ruber* | 27.5914, -113.09 | 6.47 | 289.27 ± 6.87 | 37 ± 0.99 |
| **GLM 0.1** |  |  |  |  |
| *C. horridus* | 36.7019, -91.0277 | 7.70 | 1359.95 ± 22.89 | 1413 ± 31.46 |
| *C. viridis* | 39.6319, -106.774 | 8.30 | 1031.26 ± 18.08 | 1941 ± 15.65 |
| *C. scutulatus* | 29.2044, -104.005 | 9.53 | 941.64 ± 35.86 | 716 ± 31.45 |
| *C. enyo* | 27.251, -109.01 | 42.73 | 373.51 ± 37.89 | 27 ± 3.95 |
| *C. molossus* | 28.2196, -101.748 | 10.54 | 1024.22 ± 35.94 | 1027 ± 33.79 |
| *C. basiliscus* | 22.4623, -103.673 | 18.25 | 429.28 ± 11.41 | 115 ± 7.09 |
| *C. mitchellii* | 31.3765, -111.249 | 10.20 | 611.25 ± 27.18 | 212 ± 7.61 |
| *C. tigris* | 30.6403, -109.46 | 13.05 | 277.43 ± 10.55 | 95 ± 8.89 |
| *C. adamanteus* | 30.2508, -98.7998 | 20.73 | 1363.89 ± 9.54 | 279 ± 15.43 |
| *C. atrox* | 30.179, -98.5779 | 7.97 | 1091.04 ± 17.89 | 1504 ± 55.04 |
| *C. ruber* | 27.4189, -109.524 | 33.92 | 408.32 ± 48.12 | 103 ± 14.35 |
| **GLM 0.2** |  |  |  |  |
| *C. horridus* | 36.2763, -90.9984 | 8.81 | 1275.69 ± 33.40 | 1177 ± 66.78 |
| *C. viridis* | 39.8043, -107.689 | 6.28 | 917.31 ± 19.48 | 1687 ± 54.46 |
| *C. scutulatus* | 29.1517, -104.596 | 9.70 | 692.93 ± 24.85 | 536 ± 39.97 |
| *C. enyo* | 28.3692, -110.407 | 45.42 | 292.75 ± 65.41 | 14 ± 1.27 |
| *C. molossus* | 28.0533, -102.788 | 14.61 | 838.31 ± 44.70 | 812 ± 37.28 |
| *C. basiliscus* | 22.2175, -103.671 | 16.91 | 380.59 ± 11.51 | 78 ± 7.24 |
| *C. mitchellii* | 31.2497, -111.212 | 9.79 | 516.12 ± 24.28 | 166 ± 12.87 |
| *C. tigris* | 30.5641, -109.598 | 14.33 | 256.29 ± 15.90 | 58 ± 6.46 |
| *C. adamanteus* | 29.9976, -100.443 | 29.15 | 1345.97 ± 12.01 | 197 ± 16.52 |
| *C. atrox* | 30.0486, -99.6687 | 19.22 | 994.58 ± 23.08 | 1130 ± 64.70 |
| *C. ruber* | 27.6058, -109.63 | 18.07 | 313.37 ± 10.07 | 58 ± 7.27 |
| **GLM 0.3** |  |  |  |  |
| *C. horridus* | 36.0044, -90.7087 | 33.50 | 1215.82 ± 35.46 | 1017 ± 61.79 |
| *C. viridis* | 39.9286, -108.117 | 18.99 | 865.08 ± 36.68 | 1528 ± 77.80 |
| *C. scutulatus* | 28.9746, -104.821 | 7.19 | 637.09 ± 10.05 | 419 ± 40.59 |
| *C. enyo* | 28.9963, -111.021 | 22.02 | 190.05 ± 83.42 | 11 ± 1.58 |
| *C. molossus* | 28.0876, -103.401 | 9.12 | 734.77 ± 32.90 | 693 ± 53.48 |
| *C. basiliscus* | 22.4916, -103.907 | 17.52 | 372.72 ± 15.04 | 60 ± 6.30 |
| *C. mitchellii* | 31.3615, -111.484 | 15.29 | 480.87 ± 73.21 | 134 ± 13.43 |
| *C. tigris* | 30.602, -109.405 | 36.63 | 235.44 ± 17.07 | 37 ± 13.93 |
| *C. adamanteus* | 29.7979, -101.479 | 51.96 | 1352.65 ± 13.67 | 150 ± 20.51 |
| *C. atrox* | 30.0002, -100.884 | 18.17 | 902.36 ± 38.82 | 921 ± 81.01 |
| *C. ruber* | 27.5885, -109.61 | 28.94 | 303.21 ± 19.88 | 42 ± 7.16 |
| **GLM 0.4** |  |  |  |  |
| *C. horridus* | 35.7267, -90.1057 | 49.09 | 1160.44 ± 52.01 | 907 ± 65.08 |
| *C. viridis* | 40.0407, -108.463 | 12.67 | 819.57 ± 29.33 | 1415 ± 109.43 |
| *C. scutulatus* | 28.7631, -104.76 | 9.82 | 619.34 ± 9.58 | 335 ± 43.12 |
| *C. enyo* | 28.9146, -111.06 | 25.05 | 196.66 ± 71.36 | 10 ± 2.70 |
| *C. molossus* | 28.1465, -103.654 | 9.14 | 675.52 ± 31.89 | 590 ± 59.38 |
| *C. basiliscus* | 22.5117, -103.9 | 33.15 | 358.14 ± 33.16 | 47 ± 8.21 |
| *C. mitchellii* | 31.8238, -111.869 | 11.29 | 373.09 ± 14.61 | 109 ± 14.31 |
| *C. tigris* | 29.9477, -109.22 | 54.72 | 221.56 ± 29.78 | 10 ± 2.75 |
| *C. adamanteus* | 29.6087, -102.311 | 82.82 | 1377.94 ± 38.46 | 110 ± 18.00 |
| *C. atrox* | 29.9897, -101.823 | 52.79 | 798.22 ± 73.65 | 752 ± 104.88 |
| *C. ruber* | 27.3944, -109.539 | 23.73 | 289.18 ± 30.44 | 26 ± 6.34 |
| **GLM 0.5** |  |  |  |  |
| *C. horridus* | 35.4318, -89.3415 | 29.16 | 1092.24 ± 45.47 | 818 ± 80.48 |
| *C. viridis* | 40.1148, -108.703 | 16.77 | 787.13 ± 32.23 | 1299 ± 135.27 |
| *C. scutulatus* | 28.5445, -104.662 | 14.87 | 611.50 ± 22.08 | 267 ± 51.23 |
| *C. enyo* | 29.6632, -111.163 | 31.20 | 73.56 ± 69.50 | 6 ± 1.87 |
| *C. molossus* | 28.1168, -103.714 | 10.04 | 626.41 ± 32.79 | 626.41 ± 32.79 |
| *C. basiliscus* | 22.8931, -104.278 | 19.72 | 335.55 ± 44.07 | 335.55 ± 44.07 |
| *C. mitchellii* | 32.1549, -112.208 | 19.24 | 349.21 ± 17.23 | 349.21 ± 17.23 |
| *C. tigris* | 30.5615, -108.962 | 61.55 | 138.74 ± 41.93 | 138.74 ± 41.93 |
| *C. adamanteus* | 29.4857, -105.163 | 132.15 | 1295.32 ± 136.80 | 1295.32 ± 136.80 |
| *C. atrox* | 29.9298, -102.982 | 21.47 | 660.90 ± 39.72 | 660.90 ± 39.72 |
| *C. ruber* | 26.7034, -108.672 | 55.02 | 312.72 ± 36.92 | 312.72 ± 36.92 |
