# Supplementary material for: Pleistocene Climate, Phylogeny, and Climate Envelope Models: An Integrative Approach to Better Understand Species' Response to Climate Change
Source: PLoS One. 2011 Dec 2;6(12):e28554. doi: 10.1371/journal.pone.0028554 (PMC3229599; doi:10.1371/journal.pone.0028554)
Supplement: Table S2 — Tests for the fit and stability of climate envelope models. Independent two sample t-tests for departure of modeled distributions from the known species' distribution (Model Fit) and for departure of 100 randomly subsampled models, each at 25% of the original data, from the known species' distribution (Model Stability). For model fit, the P values indicate the probability that the model is identical to the known distribution apart from chance. An asterisk indicates the model is significantly different from the known distribution. For model stability, the P value indicates the mean probability that the subsampled models are identical to the climate envelope model except by chance. Overlapping proportion (OP) is the proportion of points in the model and the known distribution. (DOC) [file pone.0028554.s002.doc]

**Table S2.**

|  | **Model Fit** | |  |  | **Model Stability** | | |
| --- | --- | --- | --- | --- | --- | --- | --- |
|  | T | DF | P | OP | T | DF | P |
| **BIOCLIM (Min-Max)** | |  |  |  |  |  |  |
| *C. horridus* | 5.51 | 1981.69 | <0.001* | 0.81 | 0.14 | 195.52 | 0.89 |
| *C. viridis* | 4.58 | 3679.43 | <0.001* | 0.72 | 0.23 | 102.76 | 0.82 |
| *C. scutulatus* | 1.24 | 784.84 | 0.21 | 0.60 | 0.10 | 145.12 | 0.92 |
| *C. enyo* | 0.54 | 20.59 | 0.59 | 0.68 | 0.02 | 192.56 | 0.99 |
| *C. molossus* | 2.43 | 1411.94 | 0.02* | 0.73 | 0.04 | 100.38 | 0.97 |
| *C. basiliscus* | 2.63 | 119.42 | 0.01* | 0.74 | 0.06 | 129.89 | 0.95 |
| *C. mitchellii* | 4.71 | 302.58 | <0.001* | 0.47 | 0.11 | 116.66 | 0.92 |
| *C. tigris* | 1.56 | 79.23 | 0.12 | 0.58 | 0.02 | 105.26 | 0.98 |
| *C. adamanteus* | 1.86 | 277.92 | 0.06 | 0.86 | 0.06 | 189.47 | 0.95 |
| *C. atrox* | 7.34 | 2390.61 | <0.001* | 0.59 | 0.17 | 101.67 | 0.87 |
| *C. ruber* | 0.41 | 78.92 | 0.68 | 0.85 | 0.00 | 149.25 | 1.00 |
| **BIOCLIM (5%-95%)** | |  |  |  |  |  |  |
| *C. horridus* | 0.91 | 1639.36 | 0.36 | 0.79 | 0.20 | 127.31 | 0.84 |
| *C. viridis* | 2.42 | 2490.75 | 0.02* | 0.82 | 0.07 | 196.89 | 0.95 |
| *C. scutulatus* | 0.53 | 681.24 | 0.60 | 0.68 | 0.12 | 194.98 | 0.90 |
| *C. enyo* | 0.04 | 18.91 | 0.97 | 0.49 | 0.02 | 193.31 | 0.98 |
| *C. molossus* | 0.62 | 1092.59 | 0.54 | 0.74 | 0.06 | 140.39 | 0.95 |
| *C. basiliscus* | 3.27 | 117.90 | 0.00* | 0.58 | 0.09 | 125.53 | 0.93 |
| *C. mitchellii* | 1.36 | 275.85 | 0.17 | 0.57 | 0.08 | 146.42 | 0.93 |
| *C. tigris* | 0.60 | 84.40 | 0.55 | 0.47 | 0.04 | 110.46 | 0.97 |
| *C. adamanteus* | 1.13 | 223.48 | 0.26 | 0.75 | 0.01 | 121.58 | 1.00 |
| *C. atrox* | 3.36 | 1407.29 | <0.001* | 0.74 | 0.07 | 172.49 | 0.94 |
| *C. ruber* | 0.15 | 65.54 | 0.88 | 0.70 | 0.07 | 137.67 | 0.95 |
| **GLM 0.1** |  |  |  |  |  |  |  |
| *C. horridus* | 8.94 | 2230.17 | <0.001* | 0.71 | 0.28 | 103.36 | 0.78 |
| *C. viridis* | 10.01 | 3253.48 | <0.001* | 0.76 | 0.12 | 102.78 | 0.91 |
| *C. scutulatus* | 4.86 | 882.25 | <0.001* | 0.52 | 0.06 | 106.11 | 0.95 |
| *C. enyo* | 3.63 | 25.25 | 0.001* | 0.05 | 0.00 | 186.49 | 1.00 |
| *C. molossus* | 8.50 | 1531.80 | <0.001* | 0.65 | 0.09 | 102.03 | 0.93 |
| *C. basiliscus* | 3.62 | 104.81 | <0.001* | 0.15 | 0.05 | 169.80 | 0.96 |
| *C. mitchellii* | 4.92 | 289.37 | <0.001* | 0.28 | 0.06 | 114.69 | 0.95 |
| *C. tigris* | 3.29 | 59.75 | 0.002* | 0.19 | 0.03 | 181.82 | 0.97 |
| *C. adamanteus* | 16.72 | 360.10 | <0.001* | 0.44 | 0.89 | 159.93 | 0.38 |
| *C. atrox* | 13.38 | 2196.64 | <0.001* | 0.64 | 0.23 | 107.01 | 0.82 |
| *C. ruber* | 4.64 | 64.74 | <0.001* | 0.09 | 0.00 | 118.81 | 1.00 |
| **GLM 0.2** |  |  |  |  |  |  |  |
| *C. horridus* | 8.59 | 1913.68 | <0.001* | 0.76 | 0.17 | 101.05 | 0.87 |
| *C. viridis* | 7.69 | 3017.93 | <0.001* | 0.80 | 0.05 | 102.26 | 0.96 |
| *C. scutulatus* | 4.01 | 728.71 | <0.001* | 0.56 | 0.01 | 113.74 | 1.00 |
| *C. enyo* | 2.40 | 23.49 | 0.02* | 0.07 | 0.01 | 137.43 | 0.99 |
| *C. molossus* | 6.40 | 1340.40 | <0.001* | 0.69 | 0.03 | 100.96 | 0.97 |
| *C. basiliscus* | 3.61 | 104.33 | <0.001* | 0.11 | 0.02 | 168.92 | 0.99 |
| *C. mitchellii* | 5.14 | 258.03 | <0.001* | 0.28 | 0.03 | 118.60 | 0.97 |
| *C. tigris* | 2.88 | 66.37 | 0.01* | 0.13 | 0.01 | 143.60 | 0.99 |
| *C. adamanteus* | 16.11 | 243.55 | <0.001* | 0.40 | 0.73 | 140.10 | 0.47 |
| *C. atrox* | 10.26 | 1893.74 | <0.001* | 0.72 | 0.14 | 103.81 | 0.89 |
| *C. ruber* | 4.48 | 63.51 | <0.001* | 0.04 | 0.05 | 171.17 | 0.96 |
| **GLM 0.3** |  |  |  |  |  |  |  |
| *C. horridus* | 7.86 | 1675.89 | <0.001* | 0.76 | 0.14 | 100.82 | 0.89 |
| *C. viridis* | 6.49 | 2878.00 | <0.001* | 0.81 | 0.01 | 99.92 | 0.99 |
| *C. scutulatus* | 3.50 | 704.66 | <0.001* | 0.57 | 0.06 | 173.95 | 0.95 |
| *C. enyo* | 2.35 | 19.04 | 0.03* | 0.08 | 0.02 | 123.22 | 0.99 |
| *C. molossus* | 5.06 | 1233.97 | <0.001* | 0.69 | 0.02 | 102.62 | 0.99 |
| *C. basiliscus* | 3.00 | 102.48 | 0.001* | 0.07 | 0.01 | 144.28 | 0.99 |
| *C. mitchellii* | 4.57 | 238.86 | <0.001* | 0.32 | 0.01 | 101.18 | 0.99 |
| *C. tigris* | 3.02 | 64.04 | 0.001* | 0.15 | 0.00 | 138.23 | 1.00 |
| *C. adamanteus* | 15.08 | 177.49 | <0.001* | 0.38 | 0.66 | 131.29 | 0.51 |
| *C. atrox* | 7.18 | 1662.25 | <0.001* | 0.77 | 0.06 | 100.70 | 0.95 |
| *C. ruber* | 4.33 | 66.63 | <0.001* | 0.03 | 0.04 | 185.83 | 0.97 |
| **GLM 0.4** |  |  |  |  |  |  |  |
| *C. horridus* | 6.66 | 1510.79 | <0.001* | 0.76 | 0.09 | 99.84 | 0.93 |
| *C. viridis* | 5.55 | 2770.97 | <0.001* | 0.82 | 0.00 | 100.44 | 1.00 |
| *C. scutulatus* | 3.55 | 673.86 | <0.001* | 0.55 | 0.07 | 178.60 | 0.94 |
| *C. enyo* | 2.24 | 19.49 | 0.04* | 0.09 | 0.02 | 131.66 | 0.98 |
| *C. molossus* | 4.45 | 1149.35 | <0.001* | 0.67 | 0.00 | 102.85 | 1.00 |
| *C. basiliscus* | 2.89 | 92.96 | 0.001* | 0.06 | 0.00 | 108.84 | 1.00 |
| *C. mitchellii* | 4.19 | 211.84 | <0.001* | 0.31 | 0.03 | 149.77 | 0.97 |
| *C. tigris* | 2.59 | 16.04 | 0.02* | 0.10 | 0.00 | 112.37 | 1.00 |
| *C. adamanteus* | 13.45 | 124.05 | <0.001* | 0.33 | 0.26 | 103.20 | 0.80 |
| *C. atrox* | 4.90 | 1459.21 | <0.001* | 0.77 | 0.02 | 99.47 | 0.99 |
| *C. ruber* | 4.11 | 60.93 | <0.001* | 0.03 | 0.03 | 146.02 | 0.97 |
| **GLM 0.5** |  |  |  |  |  |  |  |
| *C. horridus* | 5.34653 | 1388.54 | <0.001* | 0.75 | 0.08 | 100.10 | 0.93 |
| *C. viridis* | 4.86689 | 2657.61 | <0.001* | 0.82 | 0.01 | 100.19 | 0.99 |
| *C. scutulatus* | 3.67057 | 603.471 | <0.001* | 0.50 | 0.04 | 117.61 | 0.97 |
| *C. enyo* | 2.92188 | 14.0947 | 0.01* | 0.00 | 0.04 | 133.34 | 0.97 |
| *C. molossus* | 4.30045 | 1067.39 | <0.001* | 0.64 | 0.02 | 102.64 | 0.99 |
| *C. basiliscus* | 2.16473 | 69.4898 | 0.03* | 0.03 | 0.01 | 104.58 | 1.00 |
| *C. mitchellii* | 3.60425 | 190.432 | <0.001* | 0.32 | 0.04 | 136.81 | 0.97 |
| *C. tigris* | 3.05216 | 11.4589 | 0.01* | 0.00 | 0.02 | 105.77 | 0.98 |
| *C. adamanteus* | 14.2056 | 89.2834 | <0.001* | 0.23 | 0.07 | 99.33 | 0.95 |
| *C. atrox* | 2.17827 | 1304.55 | 0.03* | 0.77 | 0.00 | 100.63 | 1.00 |
| *C. ruber* | 4.15296 | 26.836 | <0.001* | 0.00 | 0.02 | 132.03 | 0.98 |
