# Supplementary figures and images for: Pleistocene Climate, Phylogeny, and Climate Envelope Models: An Integrative Approach to Better Understand Species' Response to Climate Change
Source: PLoS One. 2011 Dec 2;6(12):e28554. doi: 10.1371/journal.pone.0028554 (PMC3229599; doi:10.1371/journal.pone.0028554)

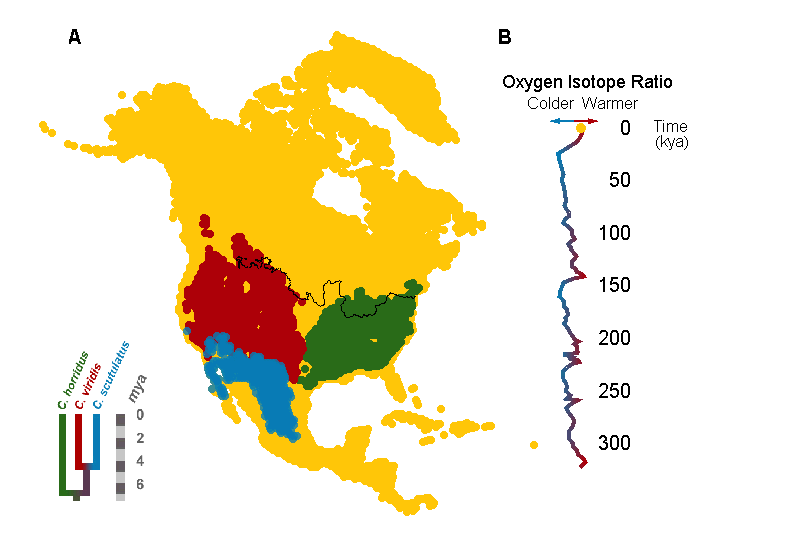

Supplement: Video S1 — Paleophylogeographic model for three rattlesnake species ( Crotalus horridus , C. viridis , and C. scutulatus ). A, Phylogeny and suitable habitat models mapped onto climatic conditions from corresponding time intervals to illustrate the effects of climate and phylogenetic changes on the distribution of suitable habitats. Phylogenetically scaled climate envelopes were projected onto isotopically scaled paleoclimate models to generate these maps. The dark gray curve represents the southern extent of glaciers during the LGM. B, Composite oxygen isotope curve for the last 320 ky inset with a yellow circle to indicate the oxygen isotope ratio and the time interval used in the model on the adjacent map. (GIF) [file pone.0028554.s003.gif]

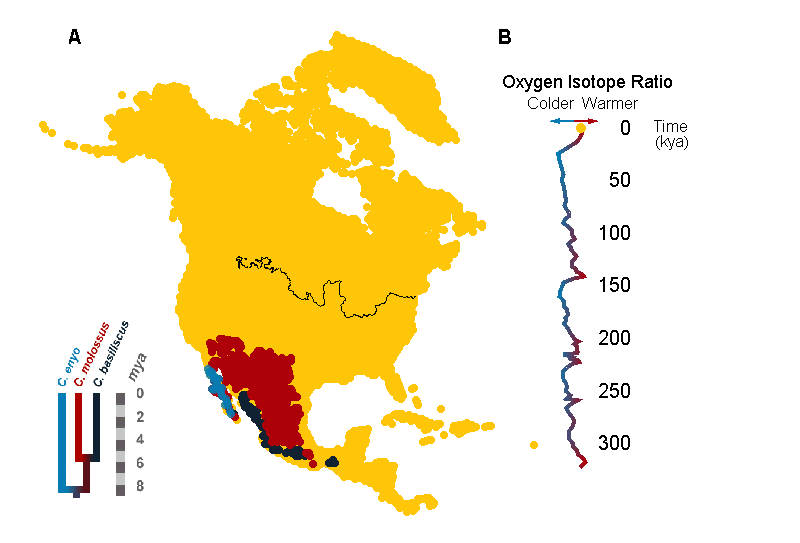

Supplement: Video S2 — Paleophylogeographic model for three rattlesnake species ( Crotalus enyo , C. molossus , and C. basiliscus ). A, Phylogeny and suitable habitat models mapped onto climatic conditions from corresponding time intervals to illustrate the effects of climate and phylogenetic changes on the distribution of suitable habitats. Phylogenetically scaled climate envelopes were projected onto isotopically scaled paleoclimate models to generate these maps. The dark gray curve represents the southern extent of glaciers during the LGM. B, Composite oxygen isotope curve for the last 320 ky inset with a yellow circle to indicate the oxygen isotope ratio and the time interval used in the model on the adjacent map. (GIF) [file pone.0028554.s004.gif]

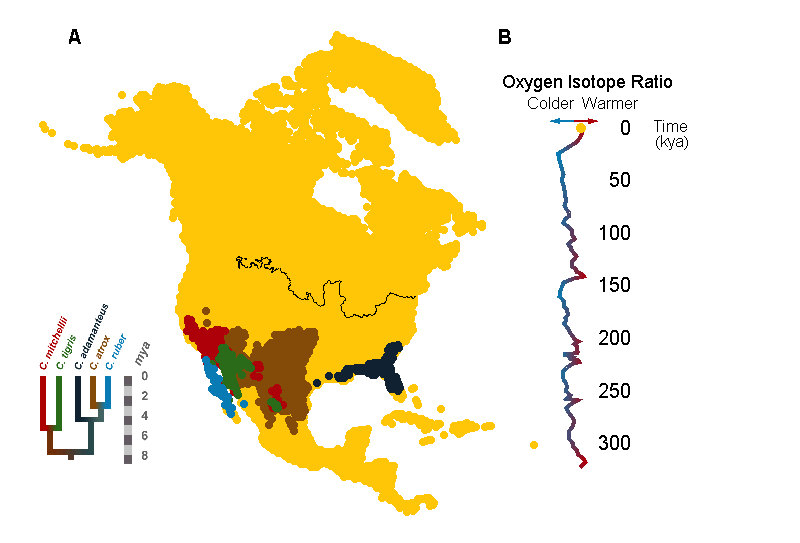

Supplement: Video S3 — Paleophylogeographic model for five rattlesnake species ( Crotalus mitchellii , C. tigris , C. adamanteus , C. atrox , and C. ruber ). A, Phylogeny and suitable habitat models mapped onto climatic conditions from corresponding time intervals to illustrate the effects of climate and phylogenetic changes on the distribution of suitable habitats. Phylogenetically scaled climate envelopes were projected onto isotopically scaled paleoclimate models to generate these maps. The dark gray curve represents the southern extent of glaciers during the LGM. B, Composite oxygen isotope curve for the last 320 ky inset with a yellow circle to indicate the oxygen isotope ratio and the time interval used in the model on the adjacent map. (GIF) [file pone.0028554.s005.gif]
